# *Bacillus thuringiensis* chimeric proteins Cry1A.2 and Cry1B.2 to control soybean lepidopteran pests: New domain combinations enhance insecticidal spectrum of activity and novel receptor contributions

**DOI:** 10.1371/journal.pone.0249150

**Published:** 2021-06-17

**Authors:** Danqi Chen, William J. Moar, Agoston Jerga, Anilkumar Gowda, Jason S. Milligan, Eric C. Bretsynder, Timothy J. Rydel, James A. Baum, Altair Semeao, Xiaoran Fu, Victor Guzov, Karen Gabbert, Graham P. Head, Jeffrey A. Haas

**Affiliations:** Bayer Crop Science, Chesterfield, Missouri, United States of America; Nigde Omer Halisdemir University, TURKEY

## Abstract

Two new chimeric *Bacillus thuringiensis* (Bt) proteins, Cry1A.2 and Cry1B.2, were constructed using specific domains, which provide insecticidal activity against key lepidopteran soybean pests while minimizing receptor overlaps between themselves, current, and soon to be commercialized plant incorporated protectants (PIP’s) in soybean. Results from insect diet bioassays demonstrate that the recombinant Cry1A.2 and Cry1B.2 are toxic to soybean looper (SBL) *Chrysodeixis includens* Walker, velvetbean caterpillar (VBC) *Anticarsia gemmatalis* Hubner, southern armyworm (SAW) *Spodoptera eridania*, and black armyworm (BLAW) *Spodoptera cosmioides* with LC_50_ values < 3,448 ng/cm^2^. Cry1B.2 is of moderate activity with significant mortality and stunting at > 3,448 ng/cm^2^, while Cry1A.2 lacks toxicity against old-world bollworm (OWB) *Helicoverpa armigera*. Results from disabled insecticidal protein (DIP) bioassays suggest that receptor utilization of Cry1A.2 and Cry1B.2 proteins are distinct from each other and from current, and yet to be commercially available, Bt proteins in soy such as Cry1Ac, Cry1A.105, Cry1F.842, Cry2Ab2 and Vip3A. However, as Cry1A.2 contains a domain common to at least one commercial soybean Bt protein, resistance to this common domain in a current commercial soybean Bt protein could possibly confer at least partial cross resistance to Cry1A2. Therefore, Cry1A.2 and Cry1B.2 should provide two new tools for controlling many of the major soybean insect pests described above.

## Introduction

Soybean, *Glycine max* (L.) is one of the most important oilseed crops globally and was cultivated on 121 million hectares (MHa) producing 334 million metric tons in 2019 [1]. Brazil is the world leader in soybean production, cultivating annually on approximately 36 MHa which amounts to almost a third of the global market. Production in Brazil, especially with a sub-tropical climate, is vulnerable to significant yield losses due to numerous factors including feeding by insects belonging to orders Lepidoptera and Hemiptera [2–4]. Important lepidopteran insect pests of soybean in Brazil include soybean looper (SBL, *Chrysodeixis includens* Walker), velvetbean caterpillar (VBC, *Anticarsia gemmatalis* Hubner), southern armyworm (SAW, *Spodoptera eridania*), black armyworm (BLAW, *Spodoptera cosmioides*) and old-world bollworm (OWB, *Helicoverpa armigera*) [5–9]. Growers historically rely on applications of chemical insecticides to control these pests which can be problematic, especially disseminating insecticides to the lower part of the canopy, where many of these insect pests feed [10, 11]. Additionally, many of the insecticides currently used to control these pests are losing efficacy due to insect resistance development [12, 13]. Therefore, since 2013, farmers have adopted the cultivation of genetically modified soybeans expressing proteins from the common soil bacterium *Bacillus thuringiensis* (Bt, Bt soybean) to control lepidopteran pests [14, 15].

The first generation Bt soybean expresses the Cry1Ac protein, which provides control of SBL, VBC and OWB [15–17]. The use of Bt soybean has fundamentally improved soybean crop protection practices and strategies, while bringing substantial environmental and economic benefits leading to a relatively rapid adoption of the globally planted Bt soybean (from 2.2 MHa in 2013/2014 to 20 MHa in 2016/2017) [18–20]. The primary threat to the durability of Bt soybean is the development of field-evolved resistance, as has been demonstrated in cotton and maize [21–24]. Therefore, soon to be commercialized second generation insect protected soybean expresses multiple Bt proteins; Cry1Ac, Cry1A.105 and Cry2Ab2, or Cry1Ac and Cry1F, providing not only SBL, VBC and OWB control but also expands the efficacy spectrum to SAW and BLAW [25–28]. However, with the anticipated rapid adoption of second-generation Bt soybeans, there is an ongoing need to identify new insecticidal proteins that can control future Bt-resistant insects in the field and provide growers with options for continued durability.

The Bt mode of action; the sequence of events leading to insect death by Bt proteins as explained in the classical model, is relatively simple, well-understood, and generally has been accepted for 40 years [29–33]. This model involves six basic steps: ingestion, proteolysis, receptor binding, membrane insertion, pore formation, and damaged epithelium leading to insect death. This well accepted model is broadly applicable to many classes of Bt proteins including three domain crystal proteins (3D-Cry), vegetative insecticidal proteins (Vip), ETX-MTX like proteins, or Bt proteins derived through domain swapping or targeted mutations [22, 34]. Therefore, new Bt proteins, particularly Cry proteins, do not require detailed studies to understand their general mode of action; but rather, distinction of the receptors they interact with during the binding step, as numerous studies have demonstrated that resistance to Bt proteins is often linked to an alteration in receptor binding [35–38]. Expression of pyramided Bt proteins with different receptor utilization, therefore, not only effectively controls insects resistant to currently available products, but also increases Bt trait durability by delaying resistance significantly longer than a single Bt trait [21, 39–41].

Here, we report the development of two new modified Bt proteins, Cry1A.2 and Cry1B.2, with toxicity against five target lepidopteran pests of soybeans, as well as corn earworm (CEW, *Helicoverpa zea* Boddie) as a surrogate species. Using the recently developed disabled insecticidal protein (DIP) assay [42], we demonstrate that Cry1A.2 and Cry1B.2 differ in receptor utilization from each other, commercially available and soon to be commercialized Bt proteins in soy including Cry1Ac, Cry1A.105, Cry1F.842, Cry2Ab2 and Vip3A, demonstrating their utility in future insect protected soybean products.

## Materials and methods

### Materials

Luria Broth (LB), agar, chloramphenicol, Q sepharose^®^, HIS-Select®, sodium carbonate, sodium chloride (NaCl), tris(hydroxymethyl)aminomethane (Tris), ethylenediaminetetraacetic acid (EDTA), bovine serum albumin (BSA), hydrochloric acid (HCl), phenylmethylsulfonyl fluoride (PMSF), benzamidine, tris(2-carboxyethyl) phosphine (TCEP), Triton X-100, polyethylene glycol 8000 (PEG8000), magnesium chloride (MgCl_2_), trypsin from bovine pancreas, and chloramphenicol were from Millipore Sigma. QuikChange^®^ kit was from Agilent Technologies. Rosetta™ 2(DE3) competent cells were from Novagen. Wizard™ classic crystallization screen was from Rigaku Reagents.

### Insecticidal proteins

Insecticidal proteins used in this study include the native and disabled counterparts of Cry1A.107, Cry1A.105, Cry2Ab2, Cry1B.2, Cry1A.2 (described below), Cry1F.842, and Vip3A. Cloning of the native Cry1A.107, Cry1A.105, Cry2Ab2, Cry1F.842 and Vip3A was carried out as reported previously [43].

### Cloning of Cry1A.2 and Cry1B.2 and their disabled counterparts

Genes encoding Cry1A.2 and Cry1B.2 were cloned as part of a domain chimeragenesis program of known Cry proteins from various classes using ordered gene assembly. To clone Cry1A.2 containing domain 1 from Cry1Ah, domain 2 from Cry1Ac, domain 3 from Cry1Ca, and the pro-toxin domain from Cry1Ac, DNA fragments encoding each individual domain bearing nucleotide extensions complementary to adjacent domains and the Type IIS restriction enzyme site, EarI, were first synthesized or PCR-amplified. After sequence verification, DNA fragments were annealed together then cloned into the Bt expression vector pMON106128, bearing the protoxin domain from Cry1Ac by ligation in the presence of EarI and T4 DNA ligase. The resulting expression plasmid of Cry1A.2 (pMON144763) contains the Cry1 promoter sigK/sigE for expression in Bt, and confers chloramphenicol resistance in both *E*. *coli* and Bt.

Similar methods as described above were employed in the cloning of the Cry1B.2 gene. DNA fragments encoding domain 1 and domain 2 of Cry1Be2 were ligated to the domain 3 fragment of Cry1Ka. DNA sequence encoding the C-terminal half of the protein extending beyond domain 3 and comprising the protoxin moiety was derived from Cry1Ab3. The resulting coding regions were cloned into the Bt expression vector pMON106128. The resulting Cry1B.2 amino acid sequence encoded on the expression plasmid pMON236926 comprises its domains 1 to 3, and the C-terminal protoxin moiety from Cry1Ab3.

DIPs were produced by introducing disabling mutations to the pore-forming domain, domain 1, of the respective 3D-Cry proteins, and to the positions involved in the oligomeric interface of the Vip3 protein by site-directed mutagenesis using the QuikChange® kit as reported previously [43]. The disabling substitutions for each insecticidal protein are: Cry1A.107 (I109C and E129C), Cry1A.105 (I109C and E129C), Cry2Ab2 (R129Q, R139Q, G119C, N123A, L156C and R160A), Cry1B.2 (A160N and N167D), Cry1A.2 (E99C and R144C), Cry1F.842 (I108C and D128C) and Vip3A (S175C and L177C).

### Expression and purification of insecticidal proteins

All insecticidal proteins were expressed in the acrystalliferous (Cry-) Bt strain EG10650 transformed with their respective expression plasmids (S1 Table) [44]. Cry protein expression was performed as reported previously with the following modifications [43]. Single colonies from the resulting transformant were isolated on LB agar supplemented with 5 μg/mL chloramphenicol at 30°C following overnight growth and used to inoculate 2.5 mL LB starter cultures containing 3 μg/mL chloramphenicol. Cultures were grown at 25°C on a rotating roller drum overnight and diluted into 500 mL Bt media containing 3 μg/mL chloramphenicol in a 2 L baffled flask and cultured at 25°C for 60 hours until verification of sporulation, crystal formation, and cell lysis by phase contrast microscopy. Crystalline insecticidal proteins and spores were harvested by centrifugation at 4°C, and then treated with 125 mL TX washing buffer containing 10 mM Tris at pH 7.5 and 0.005% Triton-X 100 supplemented with 0.1 mM PMSF for 30 min and centrifuged again. The pellet was resuspended in TX buffer and centrifuged for two additional cycles. The resulting Cry proteins, as crystal spore preparations (CSP), were used for insect diet bioassays. Cry1A.107, Cry1A.2 and their respective DIPs were also trypsin-digested after solubilization from their CSP form in 50 mM sodium carbonate (pH 11) with 5 mM TCEP, 1 mM PMSF, 1mM EDTA and 1 mM benzamidine for 1 hour, subsequent centrifugation to eliminate the insoluble fraction, and trypsinization by incubating the solubilized protein samples with trypsin at a 1/10 molar ratio at room temperature for 1.5 hours. The trypsin-resistant core of these three-domain Cry proteins (domains 1–3) was then purified on a Q Sepharose® anion exchange column. Purification of Vip3A and its DIP was conducted as reported previously [43]. Spot densitometry using BSA as standard on SDS-PAGE was used to quantitate protein samples. Expression of the native and disabled Vip3A with a N-terminal His-tag was conducted by transforming Rosetta™ 2(DE3) competent cells with the respective expression plasmids (S1 Table). The cell pellet from a 2L culture of the transformant in auto induction media was lysed and purified with HIS-Select® resin. Spot densitometry using BSA standard on SDS-PAGE was used to quantitate the protein samples.

### Structure determination of a Cry1A.2 (Y140R) variant by X-ray crystallography

The tryptic core of the Cry1A.2 (Y140R) variant (PDB: 6WPC) was used for crystallization and X-ray structure determination. Crystal leads were obtained following crystallization of a ~10 mg/ml protein solution in 96-well crystal trays prefilled with the Wizard^TM^ Classic 1&2 crystallization screen. Triangular and square plate Cry1A.2 crystals resulted from the screen condition H7 (10% PEG8000, 0.1M Tris at pH 7.0 buffer, 0.2 M MgCl_2_); from one such crystal a 3.0 Å resolution data set was collected remotely to the SER-CAT 22-ID beam line on the APS Synchrotron at Argonne National Labs. These data were reduced using the HKL package [45]. Data reduction analyses revealed the crystal to possess a c-centered, orthorhombic lattice of space group C2221, with *a* = 120.6Å, b = 225.8 Å, *c* = 239.9 Å, with all angles equal to 90 degrees. The structure was solved by the molecular replacement (MR) method using the Phaser package [46] in CCP4i [47] with a Cry1A-based structure as a phasing model. A Matthews coefficient analysis of the data suggested that there are four molecules in the crystallographic asymmetric unit, and indeed four molecules of the Cry1Ac (PDB: 4ARx)-based phasing model were located by performing successive MR. Refinement was performed using Refmac5 [48], and map-fitting was done using COOT [49]. The current structure has an R-work/R-free = 21.9%/29.2% for 120–3.0 Å data.

### Structural and comparative sequence alignment analyses

Structural alignment of Cry proteins was conducted by COOT and TM-align, respectively [49, 50]. Root Mean Square Deviation (RMSD) of superimposition along with percentage of aligned residues was calculated by COOT. The structure coordinate files of the tryptic cores of Cry1A.107, Cry1A.105, Cry2Ab2, Cry1B.2-DIP, Cry1A.2 (Y140R) and Cry1F.842 in Protein Data Bank (PDB) format were used for TM-align. Pairwise structural similarity was scaled in a TM-score ranging from 0 to 1, where a score < 0.2 indicates structurally unrelated proteins, a score > 0.5 indicates high structural homology with a shared protein fold and a score = 1.0 indicates a perfect match between structures. Pairwise sequence alignment was conducted by MUSCLE using the sequence of domain 2, domain 3, and domains 2 and 3 of the native Cry proteins as the queries, respectively [51]. Shared sequence identity in percentage was calculated from the alignment. The primary sequence of each domain was determined based on the domain boundaries in the crystal structures.

### Insect diet feeding bioassays

Insecticidal efficacy towards SBL, VBC, SAW, CEW, BLAW and OWB was evaluated in artificial diet overlay feeding bioassays. SBL, VBC and SAW eggs were provided from Bayer Crop Science (Union City, TN), CEW eggs were provided from Benzon Research (Carlisle, PA), and OWB and BLAW eggs were provided from Bayer Crop Science (Sao Paulo, Brazil). Bioassays were performed in 96-well bioassay plates with 200 μL artificial diet per well [43]. 20 μL protein samples were overlaid onto the diet surface of each well and ventilated until excess moisture dissipated. Individual wells were manually infested with neonate larva (<24 hours post-hatch), targeting one larva per well. Plates were sealed, ventilated with an insect pin and incubated for five days in a chamber at a target temperature of 27°C and a target relative humidity of 60%. Insecticidal efficacy was evaluated manually based on insect mortality or stunting. Efficacy at each concentration was calculated from 24 wells, targeting 24 insect larvae and expressed as % mortality or stunting. A stunting score reflects a delay in insect development as a response to toxicity of the insecticidal protein, where a zero score is given for insects of similar size as the negative control, (insects fed with buffer only) and scores of 1, 2 and 3 to the insects alive of 50 to 75%, 25 to 50% and <25% of the size of the negative control, respectively, based on visual observation.

Native insecticidal proteins (NIPs) of 3D-Cry proteins were administered in diet bioassays using CSP mixtures in a buffer containing 10 mM Tris, 0.005% (w/V) Triton X-100 at pH 7.5. The non-crystalline Vip3 and trypsin-treated 3D-Cry proteins were applied as soluble proteins in an alkaline buffer with 25 mM sodium carbonate at pH 10.3 and 50 mM NaCl were supplemented to stabilize proteins in buffer for bioassays on SBL, CEW, SAW, OWB and BLAW. Due to the sensitivity of VBC to NaCl, the salt concentration in the alkaline buffer with soluble protein was kept at 15 mM to avoid statistically significant background insect mortality.

### Concentration response insecticidal activities

Concentration-dependent responses of NIPs on SBL, VBC, SAW, CEW, BLAW and OWB were evaluated by insect diet overlay feeding assays. For proteins exhibiting a saturating mortality response in the tested concentration range, curves were fitted with Log_10_ of the concentration vs the normalized response equation (GraphPad Prism) for determination of LC_50_ values (1).


$$y=\frac{100}{1+10^{\left((LogLC50-X)\times HillSlope\right)}}$$ (1)

Where y is the mortality in percentage and x is log_10_ of NIP concentration. For proteins exhibiting lower than 40% mortality at the maximum concentration of 6896.5 ng/cm^2^, a stunting response was plotted as a function of NIP concentration.

### Disabled insecticidal protein (DIP) competition bioassays

DIP competition bioassays were conducted using insect diet bioassay in which a fixed concentration of NIP was pre-mixed with increasing concentrations of DIP [42]. The fixed NIP concentration was the LC_90_ or minimum concentration resulting in a stunting response score of 3 for the appropriate insect species. For DIP competition assays with trypsinized 3D-Cry NIP and DIP pairs, the NIP dosing was correspondingly adjusted to supply equivalent molar quantities of the tryptic cores in these assays due to the molecular weight decreasing approximately 50 percent from ~130 kDa to ~65 kDa after proteolysis. The maximum concentration of DIP in the DIP dilution series was at least a 25-fold molar excess to the NIP concentration, while maintaining the NIP concentration constant, as the typical challenge ratio of DIP to NIP to elicit full suppression of NIP activity between a homologous pair is 20 [42, 43]. Correspondingly, the maximum DIP concentration tested for all DIP proteins in this study was 68,965 ng/cm^2^, and the NIP concentrations were kept below 3,448 ng/cm^2^ at approximately the LC_90_ dose-response, or at approximately the MIC90 dose response for NIP’s that only showed insect stunting at this dose range (Table 1 and S2 Table).

**Table 1:** Concentration dependent insecticidal activities of Cry1B.2 and Cry1A.2 for determining the appropriate concentration in DIP assays.

NIP^a^	Insects	Mean LC50 (95% CI)^b^ (ng/cm^2^)	Mean slope ± SE^c^	sy.x^d^	df^e^	R^2^	NIP^a^ dose in DIP assays (ng/cm^2^)	Expected insect response
Cry1A.2	*C*. *includens*	11.72 (8.9 to 15.5)	1.45 ± 0.13	0.79	6	0.999	68.97	Mortality, 85–99%
	*A*. *gemmatalis*	5	32	15	5	0.903	68.97	Mortality, 85–99%
	*S*. *eridania*	14.51 (6.005 to 35.06)	1.54 ± 0.83	13.34	8	0.885	344.83	Mortality, 85–99%
	*H*. *zea*	> 6,896.5	-	-	-	-	2758.6	Stunting score of 2.5
	*H*. *armigera*	> 6,896.6	-	-	-	-	-	-
	*S*. *cosmioides*	12.26 (6.319 to 23.77)	1.16 ± 0.32	11.35	8	0.897	344.8	Mortality, 85–99%
Cry1B.2	*C*. *includens*	226.3 (191.0 to 268.1)	2.27 ± 0.27	2.36	6	0.998	1724.1	Mortality, 85–99%
	*A*. *gemmatalis*	332.9 (259.5 to 427)	-	-	-	-	68.97	Stunting score of 3
	*S*. *eridania*	94.97 (47.04 to 191.8)	2.3 ± 1.35	14.48	6	0.915	344.8	Mortality, 85–99%
	*H*. *zea*	> 6,896.6	-	-	-	-	2069	Stunting score of 3
	*H*. *armigera*	5784.0 (3332 to 10042)	1.21 ± 0.34	8.72	6	0.805	3448.3	Stunting score of 3
	*S*. *cosmioides*	866.7 (673.0 to 1116)	1.64 ± 0.28	7.01	6	0.969	1724.1	Stunting score of 3

a: native insecticidal protein.

b: mean concentration of sample that is necessary to kill 50% of larvae; CI, confidence interval.

c: standard error.

d: standard deviation of the residuals calculated by GraphPad Prism.

e: degree of freedom.

Based on one-way ANOVA and post-hoc Tukey tests (α = 0.05) (GraphPad Prism) dose-response competition assays with statistically significant loss or full suppression of insecticidal activity were scored as competition, indicating that the NIP and the DIP probe have shared cognate receptor(s). Assays with no statistically significant changes of P > 0.05 in activity were recorded as no competition. Note that each NIP:DIP pair comparison using the DIP assay was conducted twice with consistent results; only one of the replicates is shown in the respective figures.

## Results

### Production of the chimeric Cry1A.2 and Cry1B.2 proteins using domain swapping

Cry1A.2 is comprised of domain 1 from Cry1Ah (M22-D272), domain 2 from Cry1Ac (T259-E461), domain 3 from Cry1Ca (I467-D616) and the pro-toxin domain from Cry1Ac (E626-E1189). Compared to other Cry1A commercial Bt proteins, Cry1A.2 exhibited >50-fold greater insecticidal activity (LC_50_ at 14.51 ng/cm^2^) against SAW compared to Cry1A.107 and Cry1A.105, while maintaining significant efficacy on other soy pests including SBL, VBC and BLAW (Table 1 and S2 Table). Cry1A.2 exhibited no significant mortality and low to moderate stunting against CEW and OWB (Table 1 and S2 Table).

Cry1B.2 is comprised of domains 1 and 2 from Cry1Be2 (M1-I503), domain 3 from Cry1Ka2 (T504-T642) and the pro-toxin domain from Cry1Ab3 (E646-E1187). Cry1B.2 was toxic to SBL, VBC, SAW and BLAW with LC_50_ values ranging from 94.97 to 866.7 ng/cm^2^ (Table 1). In addition, Cry1B.2 had a 20-fold enhanced specific FAW activity relative to its parent scaffold, Cry1Be (LC50 values are 3,478 ng/cm^2^ vs. 67,590 ng/cm^2^, S5 and S6 Figs, respectively) compared to its Cry1B parent, as well as substantial activity on OWB with LC_50_ value of 5684 ng/cm^2^.

### Construction of insecticidal protein variants disabled in their pore-forming function

**Fig 1:**
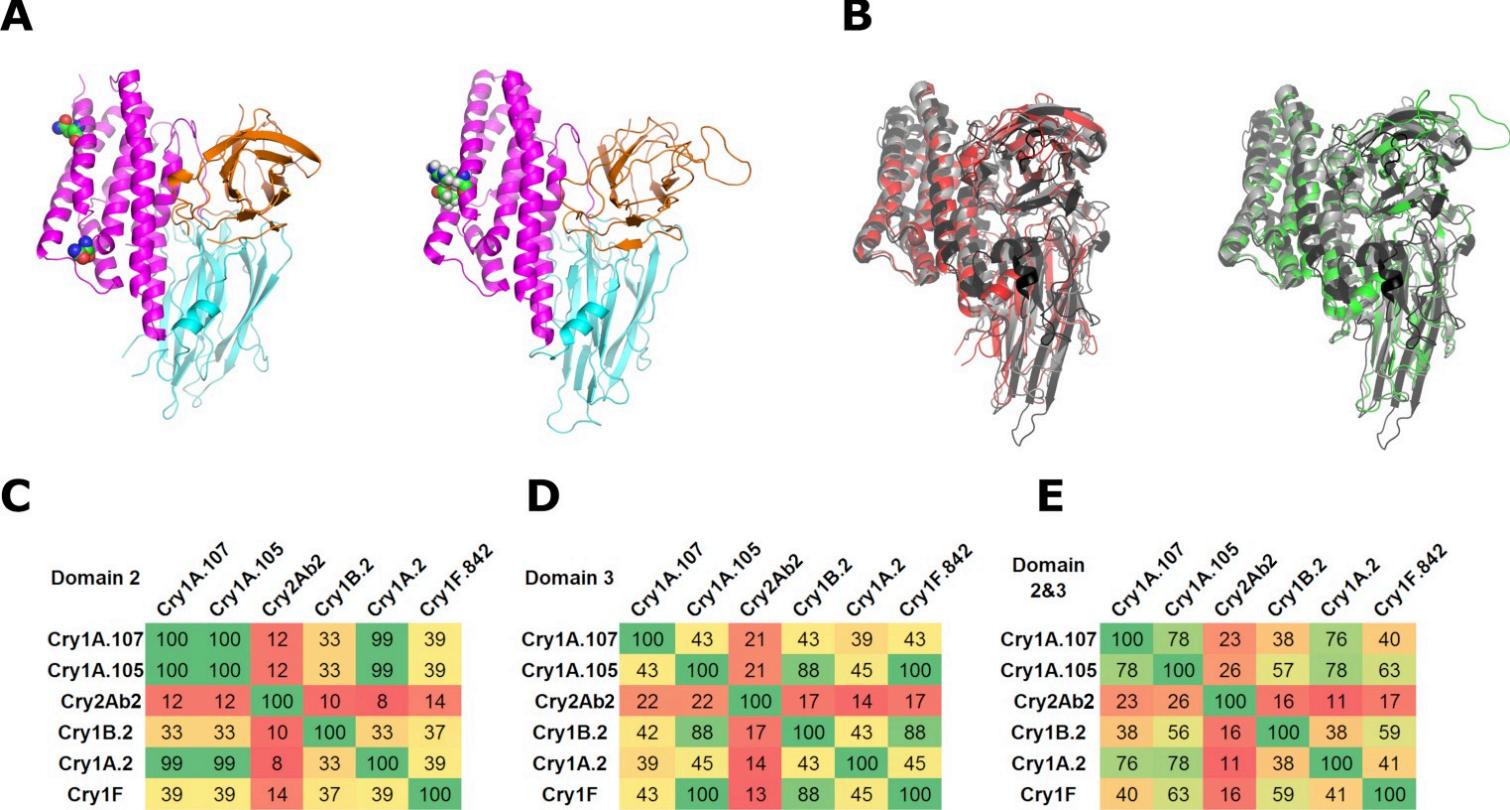
Sequence and structure relationship between *Bacillus thuringiensis* insecticidal proteins. (A) Crystal structures of the tryptic core of Cry1B.2-DIP (left panel) (PDB: 6OWK) and Cry1A.2 (right panel) (PDB: 6WPC). Domain 1, 2 and 3 of both Cry1B.2-DIP and Cry1A.2 are shown in magenta, cyan and orange, respectively. The disabling substitutions highlighted in sphere for Cry1B.2-DIP are A160N and N176D and those for Cry1A.2 are E99C and R144C. (B) Structural superimposition of Cry1Ac (PDB: 4ARX, gray) and Cry2Aa (PDB: 1I5P, black) with Cry1B.2-DIP (red, left panel) and Cry1A.2 (green, right panel), respectively. Pairwise sequence identity values between the native insecticidal proteins in domain 2 (C), domain 3 (D) and domain 2 and 3 (E), respectively. Sequence identity values colored by a gradient from blank (distant) to red (close) are calculated from pairwise sequence alignments of the insecticidal proteins by MUSCLE.

Cry1A.2 and Cry1B.2 DIP variants were produced by introducing mutations at residues in the pore-forming domain 1 (Fig 1A). The double mutant, Cry1A.2(E99C, R144C) (henceforth Cry1A.2-DIP) was identified using insect diet bioassays (left panel, Fig 1A). As Cry1B.2 and the previously reported Cry1B.868 share identical Cry1B.2 domain 1, Cry1B.2-DIP was produced by making the A160N, N167D substitutions in its domain 1 (right panel, Fig 1A) [43].

The DIP variants of Cry1A.2 and Cry1B.2, as well as all other Bt proteins in this study, were validated as *bona fide* DIPs in bioassays on six target insect pests (S3 Fig); the following observations were consistent for all *bona fide* DIP variants: 1) they were not insecticidal even at concentrations that were 20- to 100-fold higher than the LC_90_ of the corresponding native insecticidal protein (NIP), 2) when co-administered with their corresponding native insecticidal protein at a 1:1 molar ratio, no loss of insecticidal activity was recorded and 3) full suppression of NIP activity was recorded at DIP concentrations ranging from 40,000 to 70,000 ng/cm2, corresponding to a 15-25-fold DIP to NIP molar ratio.

### Structure and sequence analyses of the chimeras

Structures of domains 1–3 of Cry1A.2 (PDB: 6WPC) and Cry1B.2-DIP (PDB: 6OWK) exhibited the typical 3D-Cry protein architecture with domain 1 being comprised of an α-helical bundle, and domains 2 and 3 featuring the characteristic antiparallel β-sheets (Fig 1A). The Cry1A.2 and Cry1B.2-DIP structures can both be superimposed with Cry1Ac (PDB: 4ARX) showing RMSD (percentage of aligned residues) values of 1.2 Å (93.5%) and 1.8 Å (82.5%), respectively; and with Cry2Aa (PDB: 1I5P) where the RMSD values were 2.4 Å (73.3%) and 3.1 Å (73.0%), respectively (Fig 1B). Pairwise structural alignment by TM-align also indicated high structural homology between Cry1A.2, Cry1B.2, and the commercialized 3D-Cry proteins, as suggested by the TM-scores higher than 0.5 (S2 Fig) [50]. Together, these results demonstrated that the chimeric three-domain Cry1A.2 and Cry1B.2 proteins have the same three-dimensional architecture as Cry1A and Cry2A.

Insect receptor binding is thought to be mediated by domain 2 and/or domain 3 of 3D-Cry proteins, and therefore pairwise sequence alignment was made between the domain 2, domain 3, and domains 2 and 3 of Cry1A.2, Cry1B.2 and other 3D-Cry protein in this study. Cry1B.2 and Cry1A.2 share a low sequence identity of ~40% in domains 2 and/or 3 (Fig 1C–1E). Pairwise percent identities of Cry1B.2 and Cry1A.2 with other 3D-Cry proteins in these alignments are also low, typically less than 40% in domain 2 or 3, indicating significant sequence diversity in these two domains, with a couple of notable exceptions (Fig 1C–1E): 1) The Cry1K domain 3 of Cry1B.2 and the Cry1F domain 3 of Cry1A.105 and Cry1F.842 share 88 percent identity. Correspondingly, the sequence identity of Cry1B.2 with the two Cry1F-related proteins in both domain 2 and 3 is ~60%; 2) Due to the near identical sequence between the Cry1Ac domain 2 of Cry1A.2 and the Cry1Ab domain 2 of both Cry1A.107 and Cry1A.105, Cry1A.2 shares ~75% sequence identity in domains 2 and 3 together with Cry1A.107 and Cry1A.105 (Fig 1D and 1E).

### Differentiation of *in vivo* receptor binding preferences using the DIP competition assay

DIP pairwise competition assays were conducted between NIP:DIP homologous pairs of Cry1B.2, Cry1A.2, and all commercially exposed Bt proteins (Table 2), including Cry1A.107, Cry1A.105, Cry2Ab2, Cry1F.842 and Vip3A, as well as heterologous pairs between 1) Cry1B.2 and Cry1A.2 in competition against the DIP variants of all commercially exposed Bt proteins; 2) the same panel of exposed Bt proteins in competition with the DIP variants of Cry1B.2 and Cry1A.2. Notable exceptions include Cry1A.107 on SAW and BLAW, Cry1A.105 on SAW and Cry1A.2 on OWB (S2 Table). Due to the lack of significant insecticidal efficacy for DIP competition assays, competition assays involving either NIP or DIP of these proteins on the corresponding insects were omitted from testing.

**Table 2:** Structural class and domain classification of the native *Bacillus thuringiensis* insecticidal proteins.

Protein	Structural class	Domain Classification			
		Domain 1	Domain 2	Domain 3	Domain 4–6
Cry1A.107	three-domain Cry	1Ab	1Ab	1Ac	1Ac
Cry1A.105	three-domain Cry	1Ab	1Ab	1Fa	1Ac
Cry2Ab2	three-domain Cry	2Ab	2Ab	2Ab	N/A
Cry1B.2	three-domain Cry	1Be	1Be	1Ka	1Ab
Cry1A.2	three-domain Cry	1Ah	1Ac	1Ca	1Ac
Cry1F.842	three-domain Cry	1Fa	1Fa	1Fa	1Ac
Vip3A	Vip3	N/A	N/A	N/A	N/A

#### Cry1B.2 and Cry1A.2-NIPs vs DIPs of commercial insecticidal proteins

**Fig 2:**
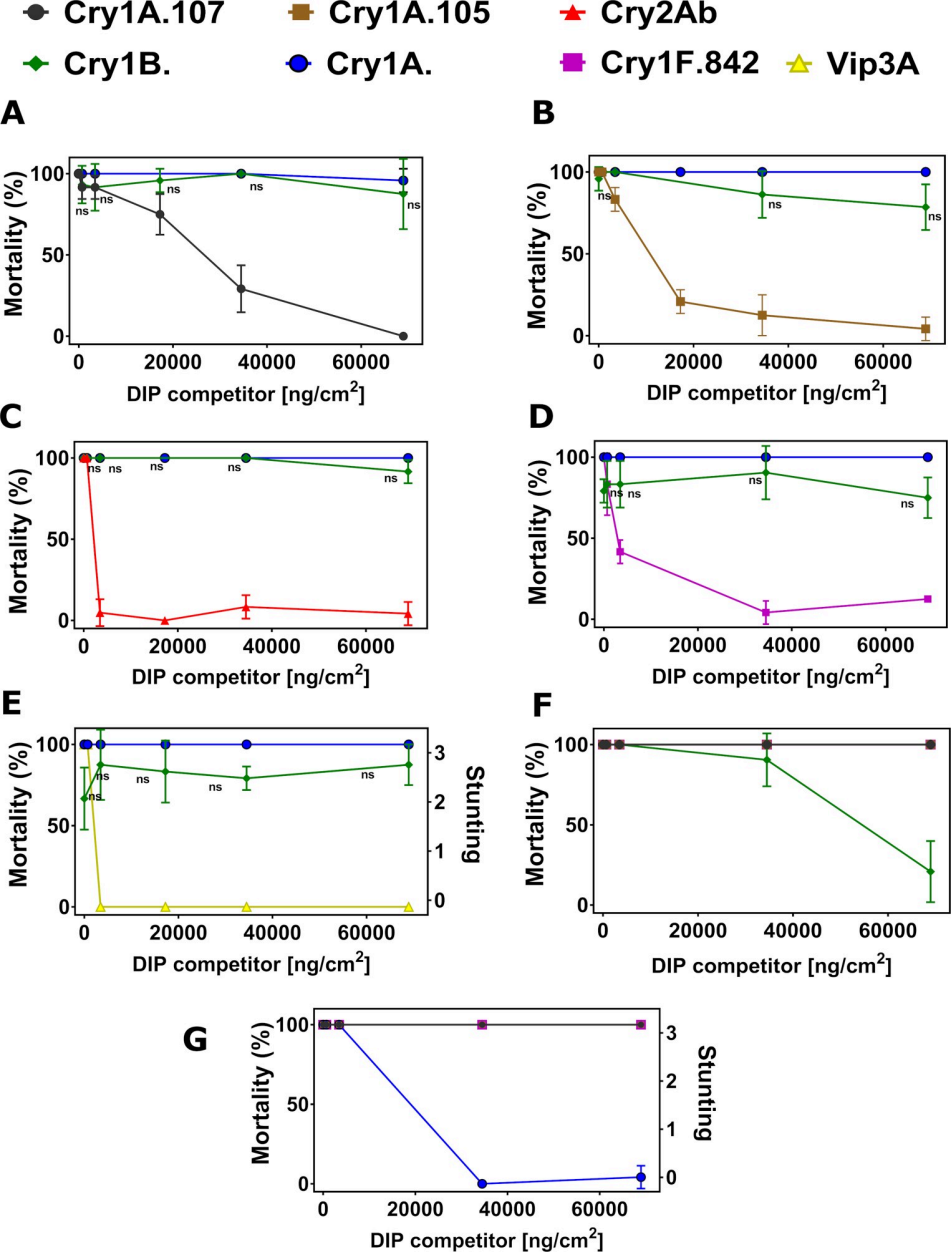
Disabled insecticidal proteins (DIPs) against native insecticidal proteins in DIP competition assays on soybean looper (SBL), *Chrysodeixis includens*. The mean of insect mortality ranging from 0 to 100 in percentage with error bars indicating the standard deviation of the mean is plotted as a function of the concentration of disabled insecticidal protein (DIP) of Cry1A.107 (A), Cry1A.105 (B), Cry2Ab2 (C), Cry1F.842 (D), Vip3A (E), Cry1B.2 (F) and Cry1A.2 (G) in the unit of ng/cm^2^.

**Fig 3:**
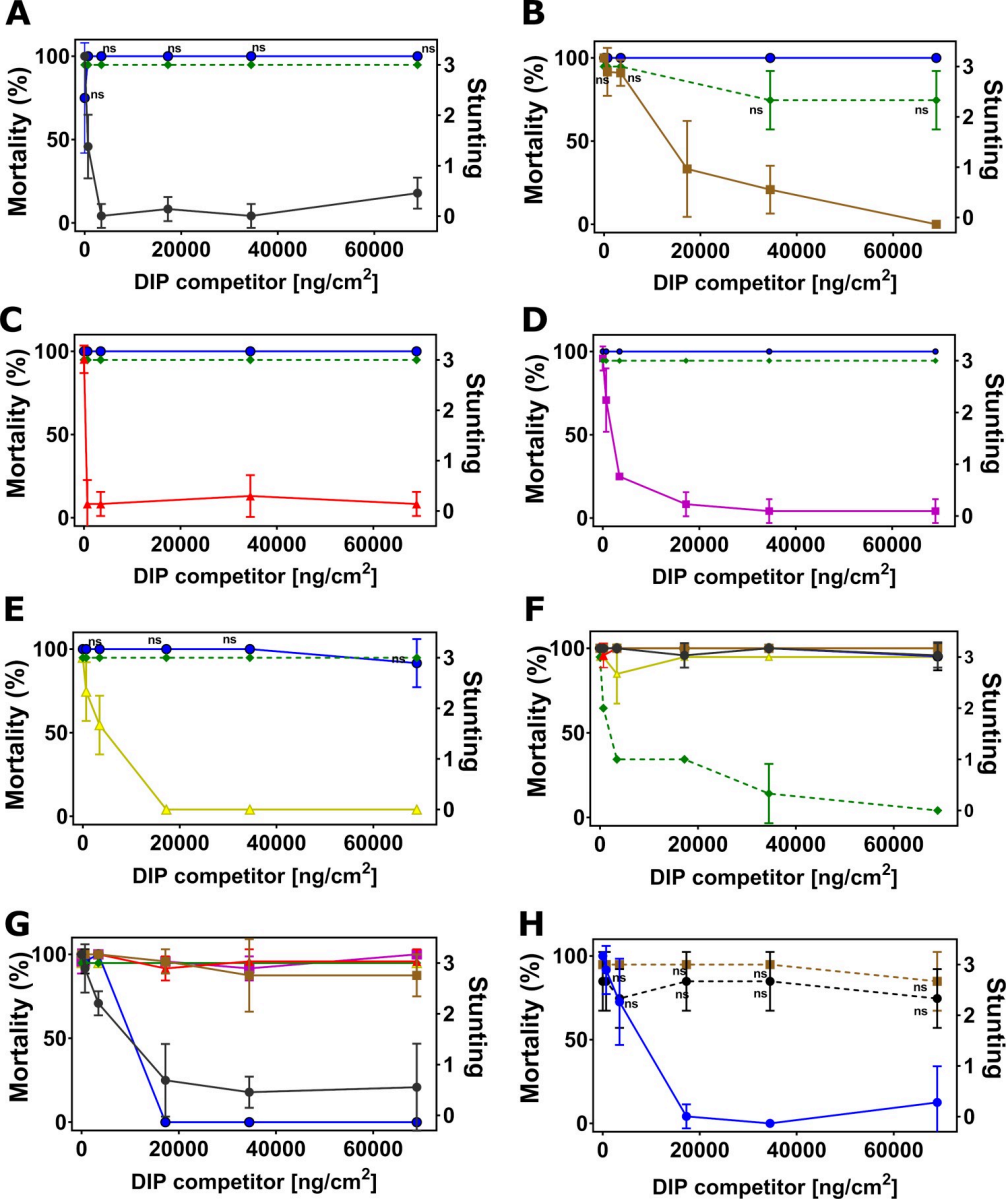
Disabled insecticidal proteins (DIPs) against native insecticidal proteins in DIP competition assays on velvetbean caterpillar (VBC), *Anticarsia gemmatalis*. The mean of insect mortality ranging from 0 to 100 in percentage or stunting response from 0 to 3 with error bar in standard deviation of the mean is plotted as a function of the concentration of disabled insecticidal protein (DIP) of Cry1A.107 (A), Cry1A.105 (B), Cry2Ab2 (C), Cry1F.842 (D), Vip3A (E), Cry1B.2 (F) and Cry1A.2 (G) in the unit of ng/cm^2^. The disabled Cry1A.2-DIP in competition against its native counterpart, Cry1A.107 and Cry1A.105 on VBC (H) with both native or disabled insecticidal proteins trypsinized. Data in mortality are connected by solid line and data in stunting response in dash line. The symbol ‘ns’ indicates that differences between the connected treatment groups show no statistical significance (P>0.05) according to 1-way ANOVA and post-hoc Tukey test (α = 0.05).

**Fig 4:**
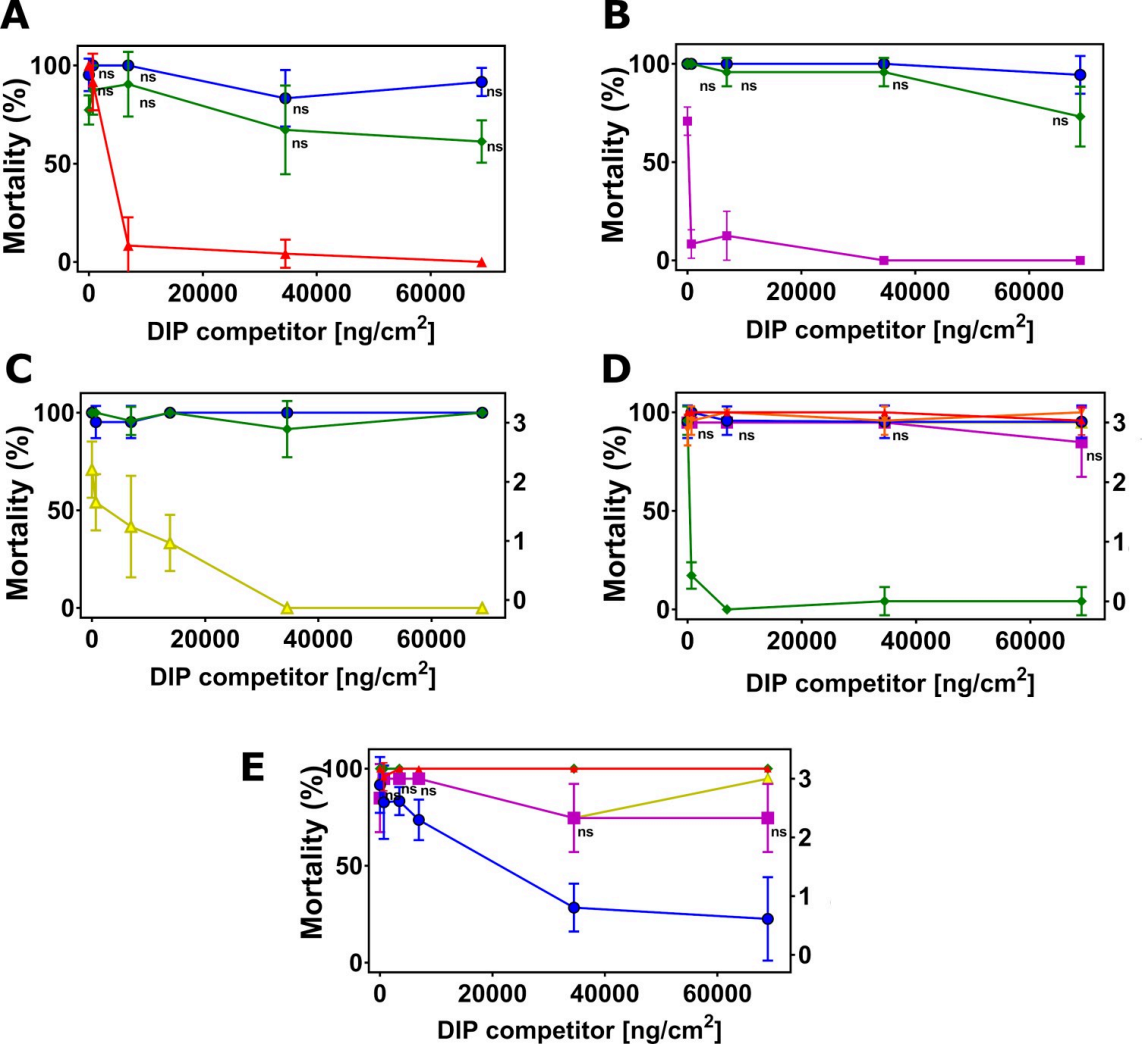
Disabled insecticidal proteins (DIPs) against native insecticidal proteins in DIP competition assays on southern armyworm (SAW, *Spodoptera eridania*). The mean of insect mortality ranging from 0 to 100 in percentage or stunting response from 0 to 3 with error bar in standard deviation of the mean is plotted as a function of the concentration of disabled insecticidal protein (DIP) of Cry2Ab2 (A), Cry1F.842 (B), Vip3A (C), Cry1B.2 (D) and Cry1A.2 (E) in the unit of ng/cm^2^. Data in mortality are connected by solid line and data in stunting response in dash line. The symbol ‘ns’ indicates that differences between the connected treatment groups show no statistical significance (P>0.05) according to 1-way ANOVA and post-hoc Tukey test (α = 0.05).

**Fig 5:**
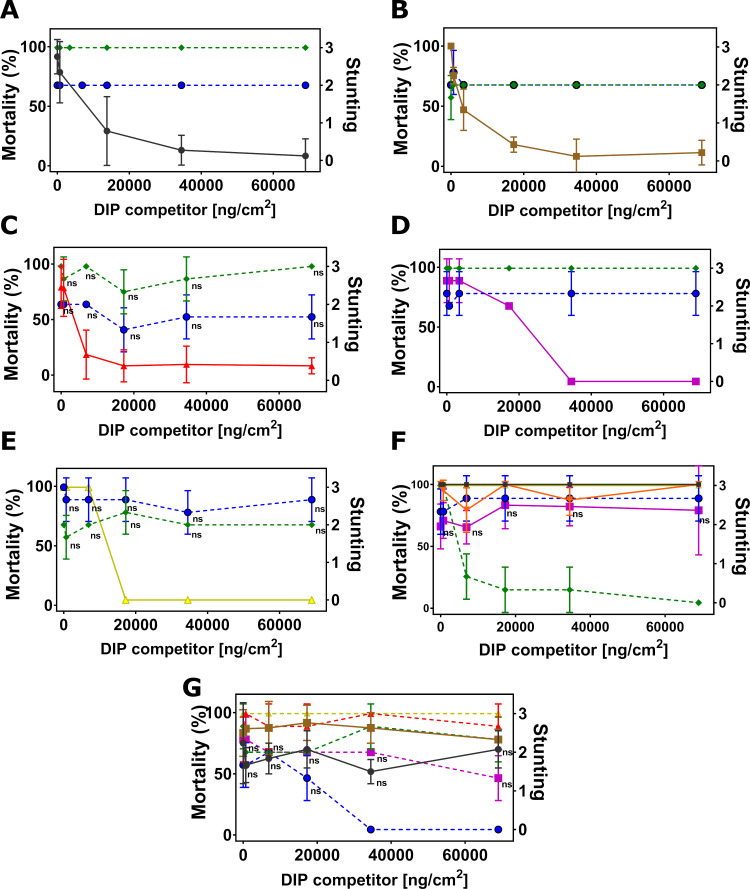
Disabled insecticidal proteins (DIPs) against native insecticidal proteins in DIP competition assays on corn ear worm (CEW, *Helicoverpa zea*). The mean of insect mortality ranging from 0 to 100 in percentage or stunting response from 0 to 3 with error bar in standard deviation of the mean is plotted as a function of the concentration of disabled insecticidal protein (DIP) of Cry1A.107 (A), Cry1A.105 (B), Cry2Ab2 (C), Cry1F.842 (D), Vip3A (E), Cry1B.2 (F) and Cry1A.2 (G) in the unit of ng/cm^2^. Data in mortality are connected by solid line and Data in stunting response in dash line. The symbol ‘ns’ indicates that differences between the connected treatment groups show no statistical significance (P>0.05) according to 1-way ANOVA and post-hoc Tukey test (α = 0.05).

**Fig 6:**
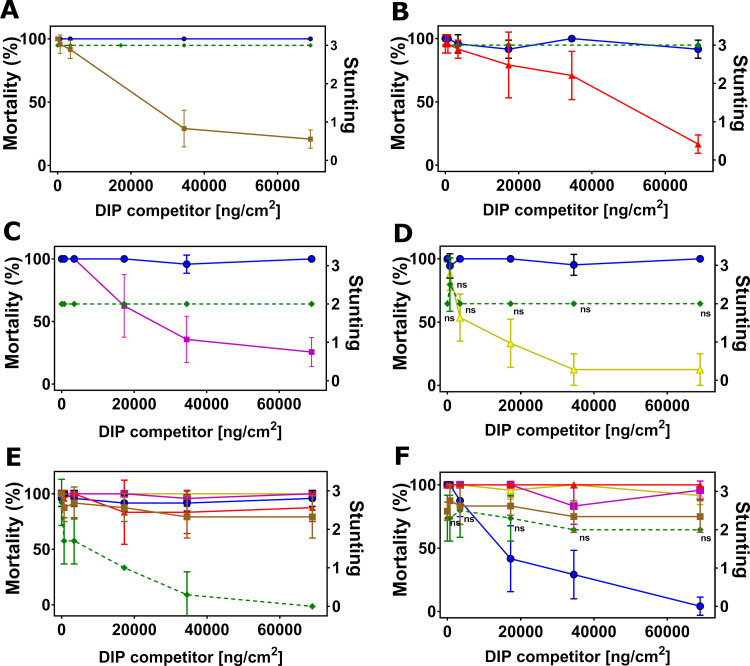
Disabled insecticidal proteins (DIPs) against native insecticidal proteins in DIP competition assays on old world bollworm (OWB), *Helicoverpa armigera*. The mean of insect mortality ranging from 0 to 100 in percentage or stunting response from 0 to 3 with error bar in standard deviation of the mean is plotted as a function of the concentration of disabled insecticidal protein (DIP) of Cry1A.107 (A), Cry1A.105 (B), Cry2Ab2 (C), Cry1F.842 (D), Vip3A (E), Cry1B.2 (F) in the unit of ng/cm^2^. The symbol ‘ns’ indicates that differences between the connected treatment groups show no statistical significance (P>0.05) according to 1-way ANOVA and post-hoc Tukey test (α = 0.05).

**Fig 7:**
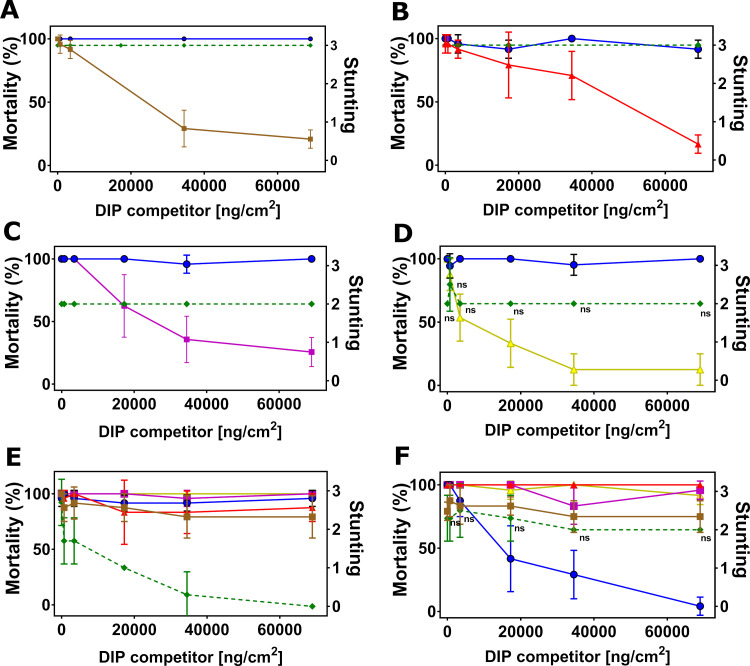
Disabled insecticidal proteins (DIPs) against native insecticidal proteins in DIP competition assays on black armyworm (BLAW), *Spodoptera cosmioides*. The mean of insect mortality ranging from 0 to 100 in percentage or stunting response from 0 to 3 with error bar in standard deviation of the mean is plotted as a function of the concentration of disabled insecticidal protein (DIP) of Cry1A.105 (A), Cry2Ab2 (B), Cry1F.842 (C), Vip3A (D), Cry1B.2 (E) and Cry1A.2 (F) in the unit of ng/cm^2^. The symbol ‘ns’ indicates that differences between the connected treatment groups show no statistical significance (P>0.05) according to 1-way ANOVA and post-hoc Tukey test (α = 0.05).

We first confirmed full homologous competition in DIP assays between NIPs and their corresponding DIPs as positive controls for all target insects. The maximum DIP concentration of 68,965 ng/cm^2^ was kept constant in all assays, as this concentration was sufficient to outcompete its parental NIP for all insect species (S3 Fig). We then conducted DIP competition assays with Cry1B.2 and Cry1A.2 NIPs against DIPs of the commercial insecticidal proteins. When challenged by increasing concentration of DIPs for Cry1A.107, Cry1A.105, Cry2Ab2, Cry1F.842 and Vip3A on SBL respectively (Fig 2A–2E), the toxicities of Cry1B.2 and Cry1A.2 NIPs at their LC_90_ were unperturbed, with no statistically significant changes (P > 0.05, 1-way ANOVA and post-hoc Tukey (α = 0.05)), in marked contrast to the homologous pairs with full inhibition in activity (S3 Fig). Similar observations were made for VBC (Fig 3A–3E), SAW (Fig 4A–4C), CEW (Fig 5A–5E), OWB (Fig 6A–6D) and BLAW (Fig 7A–7D).

#### Cry1B.2 and CryA.2-DIPs as competitors against all NIPs

In the reciprocal DIP competition bioassays between NIPs of the commercial insecticidal proteins and DIPs of the two new chimeras, the DIP variant of Cry1B.2 fully competed against its native counterpart on all six insects tested (S3 Fig), but it did not compete against the panel of commercially exposed Bt proteins even at high-dose (Figs 2F, 3F, 5F and 6F, Fig 4D, Fig 7E). Homologous competition was also observed between Cry1A.2 and its DIP variant; however, this DIP probe did not compete against any of the commercially exposed insecticidal proteins on SBL (Fig 2G), SAW (Fig 4E) and CEW (Fig 5G), and BLAW (Fig 6F) with the exception of one heterologous competition pair on VBC between Cry1A.107 and the DIP variant of Cry1A.2 that showed complete suppression of insecticidal activity (Fig 3G). We repeated the DIP assays using the trypsinized form, as opposed to the crystal protein form (CSP), of Cry1A.107, Cry1A.105, Cry1A.2 and the DIP variant of Cry1A.2. While the trypsinized Cry1A.2 DIP variant showed homologous competition against its native counterpart, it did not show significant competition against trypsinized Cry1A.107 and Cry1A.105 even in 250-fold molar excess.

## Discussion

The 3D-Cry family of insecticidal proteins, including those *in vitro*-constructed through chimeragenesis, has broad familiarity for use in control of insect pests, and has been safely deployed for decades [52, 53]. Genomic analysis of entomopathogenic bacteria previously showed evidence for the natural occurrence of chimeric 3D-Cry insecticidal proteins [34]. Also, chimeragenesis was successfully used previously to synthesize proteins with desirable spectrum and activity, (e.g. Cry1A.105). Chimeragenesis thus provides a mechanism for diversification of insecticidal proteins with novel binding preferences resulting in low cross resistance potential compared to related 3D-Cry proteins [54–57]. Here we describe two new insecticidal proteins with toxicity against key lepidopteran soybean pests with distinct binding preferences derived using chimeragenesis of the 3D-Cry family and assess their potential for cross resistance based on binding preferences using DIP assays.

Chimeric proteins described here were designed to avoid domains 2 and 3 of commercial proteins whenever possible, such as Cry1Ac, Cry1A.105, Cry1F, Cry2Ab2, and Vip3A. Following this rationale, Cry1B.2 was developed containing Cry1Be in domains 1 and 2 and Cry1Ka in domain 3 and showed substantial efficacies towards all tested insect species. Similarly, Cry1A.2 contains Cry1Ah in domain 1, Cry1Ac in domain 2 and Cry1Ca in domain 3. Although Cry1A.107 and Cry1A.2 contain Cry1Ac domain 2, domain 3 was reported to be the critical receptor binding domain in Cry1Ac for the primary soybean pest, SBL, and Cry1Ac in domain 2 is necessary to obtain the desired level of toxicity against VBC [58]. The two new chimeras adopt the well-conserved structure of 3 domain-Cry proteins. The high structural homology at the level of domain composition and folding supported the hypothesis that the two chimeras function in the same pore-formation mode-of-action as commercial Cry1 proteins such as Cry1Ab and the chimeric Cry1A.105 that maintain specificity to insects and have a long history of safe use (HOSU) [59]. Additionally, both proteins demonstrated high insecticidal activity against many of the primary target pests suggesting that these proteins would provide acceptable durability when expressed in soybean.

Loss of receptor binding site(s) has been recognized as the primary mechanism for field-evolved Bt resistance, especially when there is a relatively high dose for the particular Bt protein [35–38]. Therefore it is not surprising that binding assays are the primary *in vitro* method used to determine potential for cross resistance between insecticidal proteins [22, 30, 60–66]. We specifically chose to implement DIP assays that are based on insect diet feeding to differentiate receptor binding preferences *in vivo*, enabling a physiological assessment in target insect species with high throughput and portability [42].

The 2-way DIP competition assay results across all targeted soy lepidopteran species support distinct receptor preference of Cry1A.2 from all other insecticidal proteins evaluated. An exception is the full competition of Cry1A.107-NIP by Cry1A.2-DIP on VBC when CSP proteins were used. However, binding studies usually are conducted with the tryptic core [64, 66–69]. We found that the CSP preparations were relatively representative of the tryptic core in most cases, but not all. Given the shared receptor binding preferences between Cry1A.107 and Cry1A.105 (data not shown), it is surprising to observe full suppression of activity of Cry1A.107 but not Cry1A.105 by Cry1A.2-DIP on VBC. Also, such competition between this pair only occurred on VBC but not on any other insect species. Interestingly, the reciprocal pair between Cry1A.2-NIP and Cry1A.107-DIP or Cry1A.105-DIP in the full-length, CSP form, exhibited no competition. We wanted to explain the unanticipated competition outcome and investigated whether Cry1A.107 showed reduced insecticidal activity on VBC in the presence of a high-dose Cry1A.2 DIP variant due to receptor binding competition, or any other step in the Bt MOA model. Competition at the post-binding oligomerization step is not likely as the competition between Cry1A.107 and Cry1A.2 DIP exhibited a mass-action based competition profile (i.e. competition at high challenge ratio, Fig 3G); however, the solubilization and/or protease activation (the pre-binding steps in the MOA model) of Cry1A.107-NIP as a CSP sample may be rate-limiting on VBC when it is co-administered with a high-dose Cry1A.2-DIP. We tested this hypothesis by repeating the DIP assay using the trypsinized form of the NIP Cry1A.107-NIP and Cry1A.2-DIP proteins. We observed no competition when using these tryptic core proteins, suggesting that solubilization and/or proteolysis of the CSP forms of Cry1A-like proteins in VBC was rate limiting in the presence of Cry1A.2 DIP competitor and therefore masked the receptor utilization steps. Given the high throughput nature of the assay, such a false positive occurrence of 1 out of the total 150 competition pairs using CSP in this study does not detract from the overall value of the DIP assay in differentiating receptor preference between two insecticidal proteins. Especially, retesting of DIP competition protein pairs using the tryptic core could be carried out for result validation whenever necessary. Also, the facile use of CSP preparations in expression, purification and insect feeding bioassay is clearly more compatible to this high-volume DIP assay study compared to the use of protein tryptic cores.

Studies have demonstrated that Cry1A class proteins can recognize receptors via epitopes in domains 2 and 3 in different insect species, as exemplified by Cry1Ac binding to receptors via its domain 2 in VBC and domain 3 in SBL [58]. According to our DIP results on SBL, VBC and SPW, and the improved SAW/BLAW activity of Cry1A.2, comparisons with its Cry1A parent scaffold suggest that the Cry1C domain 3 of Cry1A.2 should be a major receptor binding contributor for these target insects either on its own, or via a receptor binding epitope nested between domains 2 and 3. Also, Cry1A.107 containing Cry1Ac domain 2 is highly active against OWB and CEW while the Cry1C protein and Cry1C domain 3-containing Cry1A.2 lacks significant activity on the two insects despite the presence of Cry1Ac domain 2. These observations are consistent with the hypothesis that the combined receptor binding domains (domains 2 and 3) in Cry1A.107 and Cry1A.2 target at least partially unique receptors across the insect species tested in this study, suggesting that Cry1A.2 would be insecticidal to these target insects even under a future Cry1A.107 resistance scenario. However, receptor interactions inherited from the parental Cry1Ac domain 2 could still be impacted by such a resistance scenario in these insects, which could lower the overall insecticidal activity of Cry1A.2.

In our series of DIP studies across all targeted insect species, the DIP probes of the commercially available Bt proteins exhibited no inhibition of Cry1B.2 toxicity, while reciprocal DIP assays with Cry1B.2 DIP also showed no inhibition of commercial protein toxicity. These results support a conclusion that there is no overlap in receptor binding between Cry1B.2 and the panel of commercially available insecticidal proteins evaluated. Interestingly, Cry1B.2 shows no overlap in receptor binding with Cry1A.105 and Cry1F.842 against all tested insect species, despite the high sequence identity of >80% in domain 3. This can be explained by receptor interactions via domain 2 of Cry1B.2, that shares low sequence identity (~30%) with the Cry1A.105 and Cry1F.842. A precedent exists for domain 2-mediated receptor binding as exemplified by the reported Cry1Ab/Ac protein interaction with a cadherin-like receptor, Bt-R1 on *Manduca sexta* [70].

Another primary advantage of using DIP assays compared to other binding assays to evaluate potential overlap in binding between candidate proteins is its portability, since one only needs to be able to carry out insect bioassays. This is especially important when binding assays need to be conducted on insect species (or strains) that are not available in a particular country, in this case, BLAW and OWB that are target pests in Brazil but do not occur in the US where the primary DIP assays are conducted. Results from Brazil for both BLAW and OWB gave similar results of no overlap in receptor binding preferences for these two pests when either Cry1A.2 or Cry1B.2 was relatively active. These results demonstrate that *in vivo* binding studies can be conducted essentially anywhere globally where insect bioassays can be conducted. This will become more important when insect control products need to be evaluated for potential cross resistance against numerous insect pests that occur in various parts of the world.

**Fig 8:**
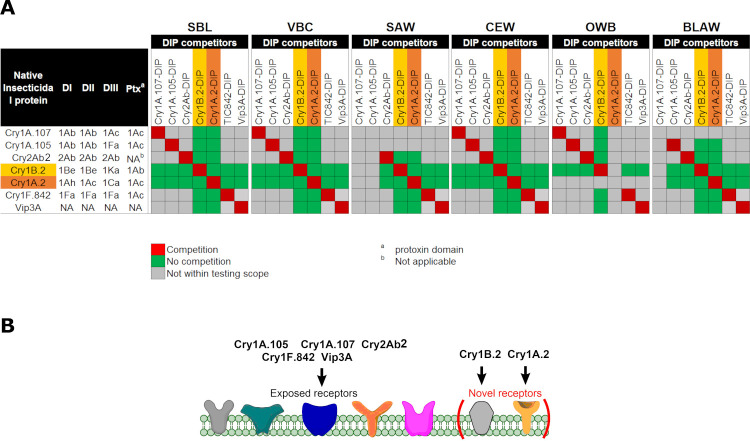
Receptor utilization relationship assessed by DIP competition assays with disabled insecticidal proteins. A) Heat map indicates the assay outcomes when a native insecticidal protein at a fixed dose was challenged by a disabled protein (DIP) as a receptor binding competitor at > 25-fold molar excess; assays with concentration dependent activity loss of native protein in the presence of DIP was recorded as competition (red), whereas those of no change or no statistically significant change in activity were scored as no competition (green). Insects tested include SBL, VBC, CEW, SAW, OWB and BLAW. The native and disabled pairs beyond the testing scope in this study are highlighted in gray. B) receptor utilization model among the insecticidal proteins in the DIP assays. According to the DIP assay results, the proteins have orthogonal receptor utilization preferences in the 6 lepidopteran insects.

A proposed model for receptor binding preferences of Cry1A.2 and Cry1B.2 based on the DIP assay results are shown in Fig 8. Taken together, Cry1A.2 and Cry1B.2 represent two new proteins with toxicity against many key lepidopteran pests of soybean, with distinct binding preferences, that could be deployed with or without currently used insecticidal proteins in soybean.

## Supporting information

S1 Fig Dose response of native insecticidal proteins in diet feeding assays on targeted insects. The mean of insect mortality in percentage ranging from 0 to 100 with error bar in standard deviation is plotted as a function of the logarithms to base 10 of insecticidal protein concentration in the unit of ng/cm^2^ on SBL (A), VBC (B), SAW (C), CEW (D), OWB (E) and BLAW (F). For proteins giving mortality <40% at the maximum dose tested on VBC (G), CEW (H), OWB (I) and BLAW (J), stunting responses also are plotted as a function of insecticidal protein concentration. See additional information on insect diet bioassay in the method section.
(DOCX)

S2 Fig Pairwise structural homology comparison of the 3-domain core of the native insecticidal protein. Pairwise structural alignment was conducted by TM-align program that ranks structural homology by TM-score that ranges from 0 (distant) to 1(close). TM score <0.2 indicates low to unrelated structural homology, while pairs with TM-score > 0.5 generally share well-conserved structural topology.
(DOCX)

S3 Fig Homologous DIP competition assays with disabled insecticidal proteins (DIP) against the corresponding native insecticidal protein counterparts. Native insecticidal proteins were competed against increasing concentrations of their corresponding disabled insecticidal protein variants on SBL (A), VBC (B), SAW (C), CEW (D), OWB (E) and BLAW (F).
(DOCX)

S4 Fig Concentration dependent insecticidal activities of trypsinized Cry1A.105 and Cry1A.107 on VBC. (DOCX)

S5 Fig Concentration dependent insecticidal activity of Cry1B.2. (DOCX)

S6 Fig Concentration dependent insecticidal activity of Cry1Be. (DOCX)

S1 Table Expression plasmids for native and disabled insecticidal proteins used in this study. (DOCX)

S2 Table Concentration dependent insecticidal activities of the commercially available insecticidal proteins in insect diet feeding assays on SBL, VBC, SAW and CEW. (DOCX)
