# Supplementary material for: Bacillus thuringiensis chimeric proteins Cry1A.2 and Cry1B.2 to control soybean lepidopteran pests: New domain combinations enhance insecticidal spectrum of activity and novel receptor contributions
Source: PLoS One. 2021 Jun 17;16(6):e0249150. doi: 10.1371/journal.pone.0249150 (PMC8211277; doi:10.1371/journal.pone.0249150)
Supplement: S1 Fig — The mean of insect mortality in percentage ranging from 0 to 100 with error bar in standard deviation is plotted as a function of the logarithms to base 10 of insecticidal protein concentration in the unit of ng/cm2 on SBL (A), VBC (B), SAW (C), CEW (D), OWB (E) and BLAW (F). For proteins giving mortality <40% at the maximum dose tested on VBC (G), CEW (H), OWB (I) and BLAW (J), stunting responses also are plotted as a function of insecticidal protein concentration. See additional information on insect diet bioassay in the method section. (DOCX) [file pone.0249150.s001.docx]

**
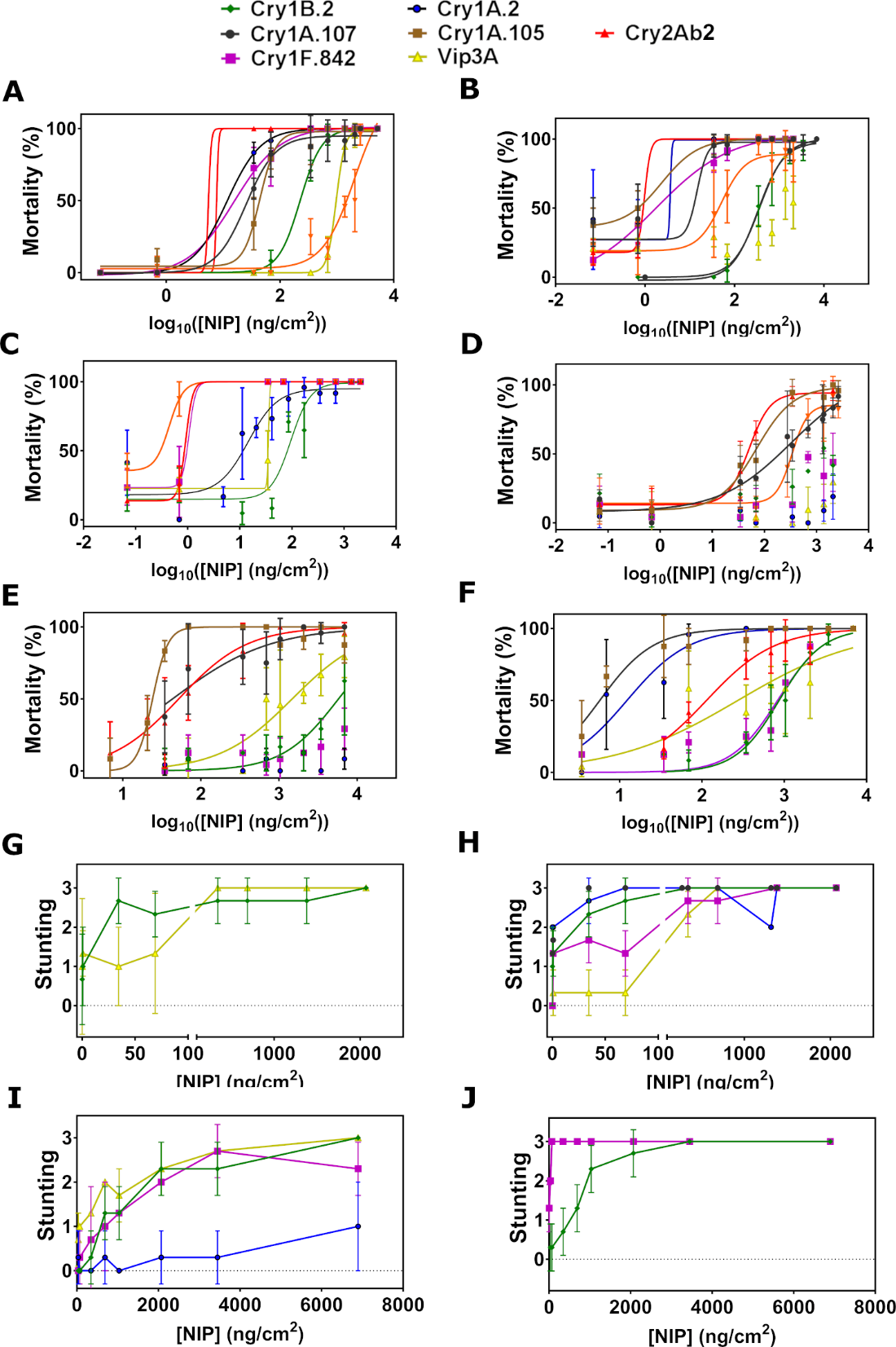
**

**S1 Fig.** **Dose response of native insecticidal proteins in diet feeding assays on targeted insects.** The mean of insect mortality in percentage ranging from 0 to 100 with error bar in standard deviation is plotted as a function of the logarithms to base 10 of insecticidal protein concentration in the unit of ng/cm^2^ on SBL (A), VBC (B), SAW (C), CEW (D), OWB (E) and BLAW (F). For proteins giving mortality <40% at the maximum dose tested on VBC (G), CEW (H), OWB (I) and BLAW (J), stunting responses also are plotted as a function of insecticidal protein concentration. See additional information on insect diet bioassay in the method section.
