# Supplementary material for: Bacillus thuringiensis chimeric proteins Cry1A.2 and Cry1B.2 to control soybean lepidopteran pests: New domain combinations enhance insecticidal spectrum of activity and novel receptor contributions
Source: PLoS One. 2021 Jun 17;16(6):e0249150. doi: 10.1371/journal.pone.0249150 (PMC8211277; doi:10.1371/journal.pone.0249150)
Supplement: S2 Fig — Pairwise structural alignment was conducted by TM-align program that ranks structural homology by TM-score that ranges from 0 (distant) to 1(close). TM score <0.2 indicates low to unrelated structural homology, while pairs with TM-score > 0.5 generally share well-conserved structural topology. (DOCX) [file pone.0249150.s002.docx]

**
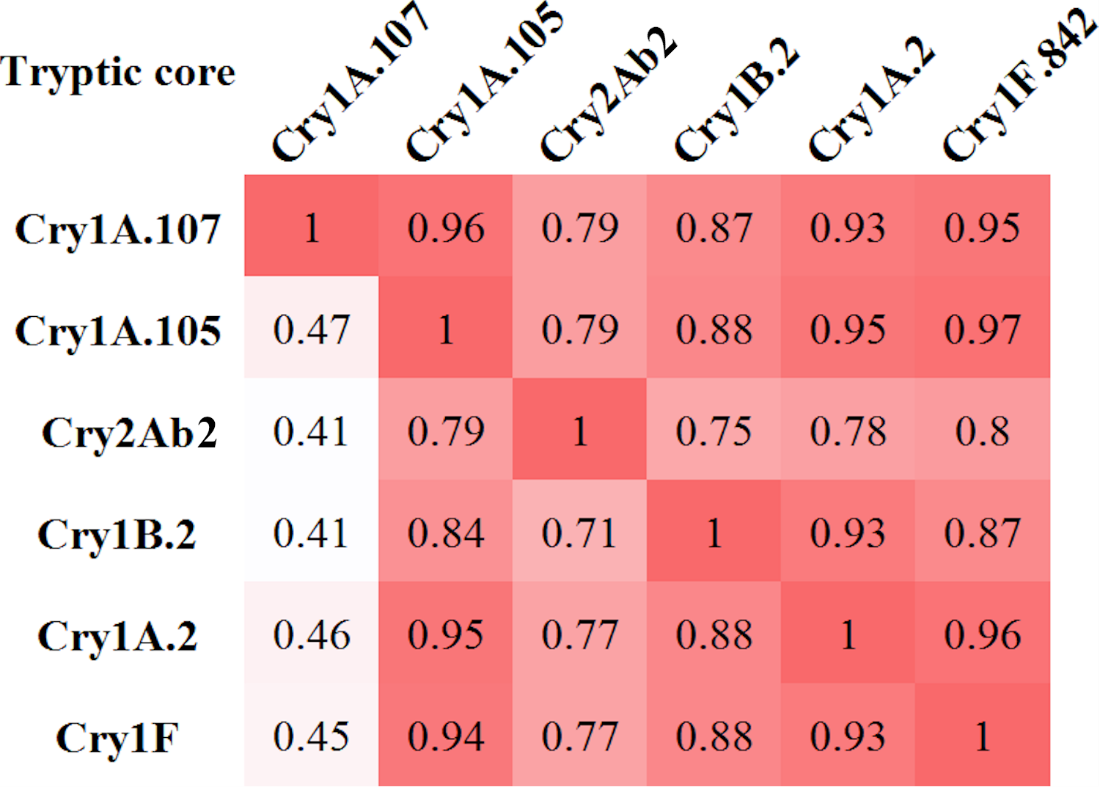
**

**S2 Fig. Pairwise structural homology comparison of the 3-domain core of the native insecticidal protein.** Pairwise structural alignment was conducted by TM-align program that ranks structural homology by TM-score that ranges from 0 (distant) to 1(close). TM score <0.2 indicates low to unrelated structural homology, while pairs with TM-score > 0.5 generally share well-conserved structural topology.
