# Supplementary material for: Bacillus thuringiensis chimeric proteins Cry1A.2 and Cry1B.2 to control soybean lepidopteran pests: New domain combinations enhance insecticidal spectrum of activity and novel receptor contributions
Source: PLoS One. 2021 Jun 17;16(6):e0249150. doi: 10.1371/journal.pone.0249150 (PMC8211277; doi:10.1371/journal.pone.0249150)
Supplement: S3 Fig — Native insecticidal proteins were competed against increasing concentrations of their corresponding disabled insecticidal protein variants on SBL (A), VBC (B), SAW (C), CEW (D), OWB (E) and BLAW (F). (DOCX) [file pone.0249150.s003.docx]

**
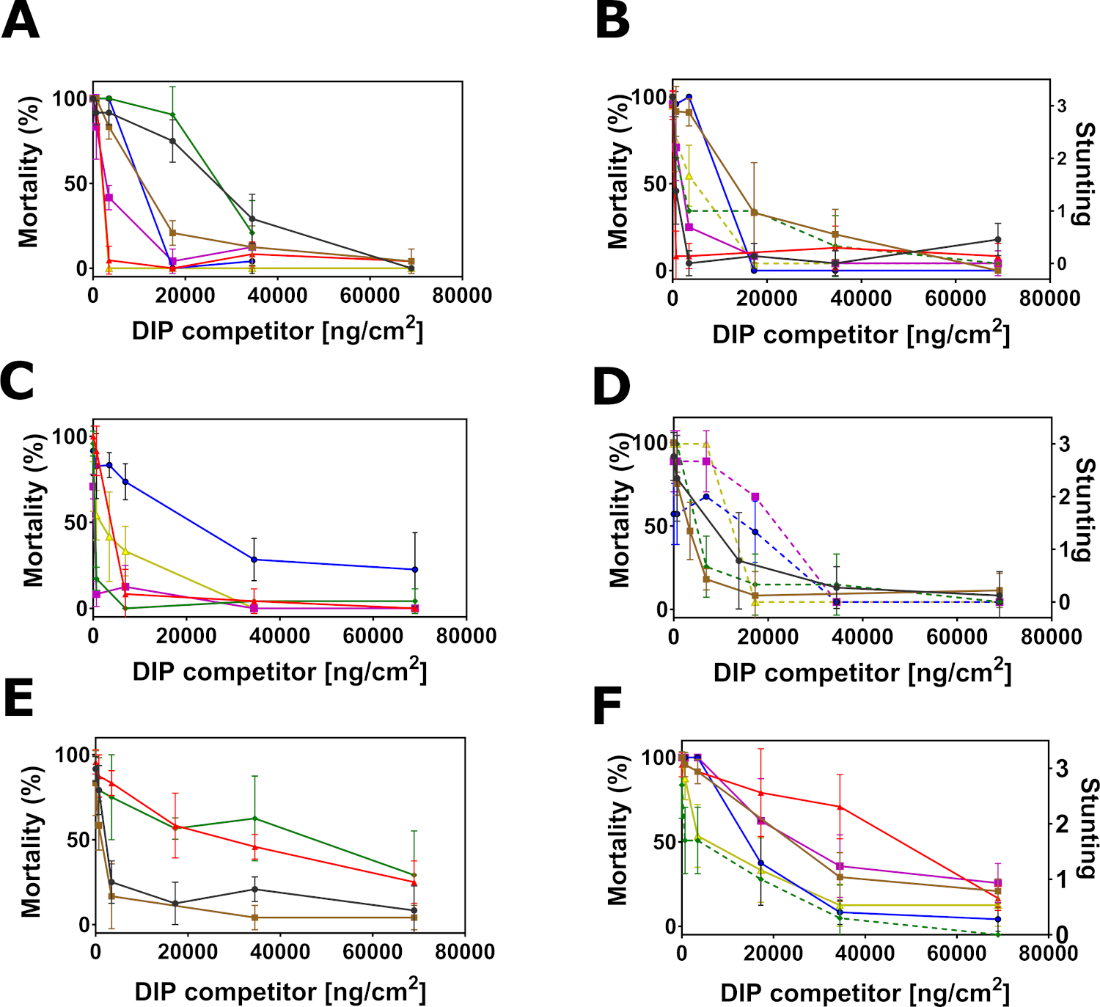
**

**S3 Fig. Homologous DIP competition assays with disabled insecticidal proteins (DIP) against the corresponding native** insecticidal protein counterparts. Native insecticidal proteins were competed against increasing concentrations of their corresponding disabled insecticidal protein variants on SBL (A), VBC (B), SAW (C), CEW (D), OWB (E) and BLAW (F).
