# Supplementary material for: Bacillus thuringiensis chimeric proteins Cry1A.2 and Cry1B.2 to control soybean lepidopteran pests: New domain combinations enhance insecticidal spectrum of activity and novel receptor contributions
Source: PLoS One. 2021 Jun 17;16(6):e0249150. doi: 10.1371/journal.pone.0249150 (PMC8211277; doi:10.1371/journal.pone.0249150)
Supplement: S4 Fig — (DOCX) [file pone.0249150.s004.docx]

**S4 Fig. Concentration dependent insecticidal activities of trypsinized Cry1A.105 and Cry1A.107 on VBC**
